# Floral Nectary Morphology and Proteomic Analysis of Nectar of *Liriodendron tulipifera* Linn.

**DOI:** 10.3389/fpls.2016.00826

**Published:** 2016-06-14

**Authors:** Yanwei Zhou, Meiping Li, Fangfang Zhao, Hongguang Zha, Liming Yang, Ye Lu, Guangping Wang, Jisen Shi, Jinhui Chen

**Affiliations:** ^1^Key Laboratory of Forest Genetics and Biotechnology, Ministry of Education, Nanjing Forestry UniversityNanjing, China; ^2^Co-Innovation Center for Sustainable Forestry in Southern China, College of Forestry, Nanjing Forestry UniversityNanjing, China; ^3^College of Life and Environment Science, Huangshan UniversityHuangshan, China; ^4^School of Life science, Huaiyin Normal UniversityHuai’an, China

**Keywords:** *Liriodendron tulipifera*, morphology, nectar protein, proteomics, defense

## Abstract

Nectar is a primary nutrient reward for a variety of pollinators. Recent studies have demonstrated that nectar also has defensive functions against microbial invasion. In this study, the *Liriodendron tulipifera* nectary was first examined by scanning electron microscopy, and then the nectar was analyzed by two-dimensional gel electrophoresis and liquid chromatography–tandem mass spectrometry, which led to identification of 42 nectar proteins involved in various biological functions. Bioinformatic analysis was then performed on an identified novel rubber elongation factor (REF) protein in *L. tulipifera* nectar. The protein was particularly abundant, representing ∼60% of the major bands of 31 to 43 kDa, and showed high, stage-specific expression in nectary tissue. The REF family proteins are the major allergens in latex. We propose that REF in *L. tulipifera* nectar has defensive characteristics against microorganisms.

## Introduction

Nectar secretion is an evolutional adaptation of many angiosperms to attract pollinators especially flying insects—for outcrossing, and nectar serves as a nutrient reward and energy source for pollinators (Nicolson and Thornburg, 2007a). Nectar is a very complex solution that is secreted by a specialized organ called the nectary. The secretion of nectar begins when flowers blossom and is usually under developmental control (Carter et al., 1999). After pollination, the nectar is frequently resorbed (Búrquez and Corbet, 1991). Floral nectar is rich in carbohydrates and amino acids (Zha et al., 2012). Other substances such as organic acids (Baker and Baker, 1975), glycosides (Roshchina and Roshchina, 1993), vitamins (Griebel and Hess, 1940), alkaloids (Deinzer et al., 1977) and flavonoids (Rodriguez-Arce and Diaz, 1992) are also present at low concentrations.

Evidence from >1500 species of flowering plants has demonstrated that the occurrence of amino acids in nectar is universal among nectariferous plants (Baker and Baker, 1986). Amino acids in nectar play a role in attracting protectors to protect plant (LANZA, 1991). Some essential amino acids in nectar from a particular flower is a crucial reason that pollinators remain changeless despite the nectar robbers and low nectar volumes (Maloof and Inouye, 2000; Petanidou et al., 2006). Furthermore, higher concentrations of amino acids in the robbed flower nectar from damaged tissues may be another reason (Camargo et al., 1984).

Proteins have also been detected in nectar, and it is thought that these proteins supply nectar consumers with organic nitrogen (Heil, 2011). Previous studies have revealed two other primary functions of nectar proteins: defense against microorganisms (Carter et al., 1999; Carter and Thornburg, 2004a; Harper et al., 2010) and post-secretory hydrolysis of nectar sugars into fructose and glucose for protectors (Heil et al., 2005). Proteins in flower nectar of ornamental tobacco (*Nicotiana langsdorffii* ×*N. sanderae*) nectar appear to protect the nectar from microbial infestation through the nectar redox cycle (Carter and Thornburg, 2004a; Carter et al., 2006, 2007; Park and Thornburg, 2009). The pathogenesis-related proteins chitinase, glucanases, and thaumatin-like proteins have been identified in nectar (Heil, 2011), as have GDSL lipase and Nectarin IV, which have been suggested to have antimicrobial functions (Kram et al., 2008; Harper et al., 2010). Thus, most of the characterized nectar proteins seem to play a role in protecting nectar against microorganisms. Although it has been some time since the presence of proteins in nectar was reported, there have been few comprehensive analyses of nectar proteins (Nepi et al., 2012).

*Liriodendron tulipifera*, also called tulip tree or yellow poplar, is a relict plant and one of only two species in this genus. *L. tulipifera* has one of the largest natural ranges of tree species of the eastern United States (Harlow and Harrar, 1969; Little, 1979). *L. tulipifera* is considered a basal angiosperm, and its floral and other structural features put it at an ideal phylogenetic position for comparative studies of the evolution of biological processes in land plants (Hunt, 1998; De Craene et al., 2003). Moreover, *L. tulipifera* is also a nectariferous plant (Liang et al., 2007). In one season, an *L. tulipifera* tree less than 20 years old can yield 3.6 kg of nectar (Beck and Sims, 1983). However, the structure of the nectary and proteins in nectar of this species are poorly understood.

Proteomics is a large-scale analysis of proteins in cell and organism has become a very important analysis method for protein characterisation (Pandey and Mann, 2000). Since two-dimensional polyacrylamide gel electrophoresis was developed for separating proteins in O’Farrell (1975) and higher resolution protein separation technologies incorporated later (Chen and Harmon, 2006), plant proteomics has largely been used to characterize responses to abiotic stress (Kosova et al., 2011) and in developmental (Hochholdinger et al., 2006; Kaufmann et al., 2011) and secretion (Agrawal et al., 2010) processes. These proteome analyses have often focused on changes at the subcellular level, such as in plastids, mitochondria, the endoplasmic reticulum, and the cell wall and membranes (Mahon et al., 2000; Canovas et al., 2004; Lee et al., 2012).

In this study, scanning electron microscopy (SEM) was used to elucidate the mechanism by which *L. tulipifera* secretes nectar and to characterize the nectary structure. Two-dimensional (2-D) gel electrophoresis and nano LC-MS/MS were applied to identify proteins in *L. tulipifera* flower nectar. Bioinformatics revealed potential functions of the proteins in nectar, and we identified a rubber elongation factor (REF) protein that we speculate may play a role in defense against microorganisms.

## Materials and Methods

### Floral Nectar Collection, pH and Protein Content Determination

Raw nectar of *L. tulipifera* L. (South Carolina accession) was collected from nearly opened flowers (pre-pollination stage) of an adult tree with a sterile pipette in a provenance trial plantation located in Xiashu, Jiangsu province (119°13′20′′E, 32°7′8′′N) at 6:00–7:00 am in May of 2014. The floral nectar samples were immediately frozen in liquid nitrogen until use.

The pH and protein content of fresh *L. tulipifera* nectar were determined according to Zha (Zha et al., 2013) and Bradford (Bradford, 1976), using bovine serum albumin as the standard. The averages of triplicate total protein content measurements are presented.

### Morphology Observation by SEM

The secreting flowers were fixed in 2.5% (v/v) glutaraldehyde buffered with 0.2 M sodium phosphate buffer (pH 7.2) for SEM. The samples were then post-fixed in 1% (w/v) osmium tetroxide for 1 h and then washed three times in the buffer. The samples were dehydrated in a graded alcohol series and then examined in a Quanta 200 environmental scanning electron microscope at an accelerating voltage of 20 kV (FEI, USA; Zimmermann et al., 2007).

### *L. tulipifera* Nectar Proteins (Nectarines) Separated by 1-D Gel Electrophoresis

Because there were no previous data on nectar proteins of *L. tulipifera*, we first identified proteins in the *L. tulipifera* nectar samples. Before electrophoresis, the samples were concentrated and purified with Amicon Ultra 3K centrifugal filter devices (Millipore, USA). The concentrated *L. tulipifera* nectar (5 μl, ∼10 μg total protein) was boiled in 2× sample buffer for 5 min then analyzed by SDS-PAGE (12.5% acrylamide gel) as described by Laemmli (Laemmli, 1970) with protein molecular weight markers (Bio-Rad, USA). Proteins were visualized by staining with Coomassie Brilliant Blue R-250.

### 2-D Gel Electrophoresis of *L. tulipifera* Nectarines and In-Gel Trypsin Digestion

For further high-resolution identification of nectarines for which the isoelectric point (pI) had not been determined, 2-D gel electrophoresis was carried out with wide range (pH 3–10) linear gradient IPG strips. The concentrated and purified *L. tulipifera* nectar was mixed with rehydration buffer containing 8 M urea, 4% CHAPS, 0.5% pH 3–10 linear IPG buffer and 14 mM dithiothreitol, and the mixture was subjected to isoelectric focusing using Ettan IPGphor III and 13 cm ReadyStrip IPG Strips (GE Healthcare, USA) with an immobilized pH gradient of 3–10 using the following settings: 12 h at 0 V, 2 h at 100 V, 1.5 h at 250 V, 1 h at 500 V, 1.5 h at increasing voltage from 2000 to 8000 V and 1.5 h at 8000 V.

Prior to second-dimension separation, the focused IPG strips were first equilibrated in 10 ml of equilibration buffer [6 M urea, 30% v/v glycerol, 75 mM Tris-HCl (pH 8.8), 2% (w/v) SDS] containing 1% DTT, then 2.5% iodoacetamide instead of DTT in the second equilibration, 15 min each at room temperature on a shaker. The equilibrated strips were transferred to the wells of 12.5% polyacrylamide gels for second-dimension electrophoresis. The wells were sealed with 0.5% agarose. SDS-PAGE was performed using the Ettan DALT *six* unit (GE Healthcare, USA) until the bromophenol blue dye front reached the bottom of the gel. After SDS-PAGE separation, the gels were stained with silver nitrate and scanned with Image Scanner (GE Healthcare, USA).

The excised protein spots were washed twice with ultrapure water and destained with 30 mM K_3_Fe(CN)_6_/100 mM NaS_2_O_3_ (1:1, v/v) for 20 min. Gel fragments were then dehydrated with 50% acetonitrile followed with 100% acetonitrile. The dried gel fragments were incubated with trypsin (20 μg/μl, Promega, USA) for 5 min at 4°C then covered and incubated with 25 mM NH_4_HCO_3_ in 10% acetonitrile at 37°C overnight (∼14 h) then extracted twice with 50 μl of 50% acetonitrile/5% trifluoroacetic acid (v/v) for 1 h. The extracted peptides were dried by freeze dryer for nanoLC-MS/MS.

### LC-MS/MS Analysis

The dried peptides were redissolved in 0.1% formic acid (v/v) and analyzed with a nano-HPLC-MS system (Easy-nLC 1000; Thermo Fisher Scientific, CA), which was coupled to an LTQ-Orbitrap XL instrument (Thermo Fisher Scientific, Bremen, Germany; LTQ, linear trap quadrupole) equipped with a nanoelectrospray interface operated in positive ion mode. All peptides were passed through a μ-Precolumn (C18, 3 μm, 100 Å, 75 μm × 2 cm) before being separated with a reverse phase column (C18, 3 μm, 100 Å, 50 μm × 15 cm, all nanoViper, Thermo Fisher Scientific, CA). The mobile phases consisted of a gradient of solvent A (0.1% formic acid) to solvent B (0.1% formic acid in acetonitrile) run for 107 min per gel spot as follows: 530% B (0–20 min); 30–90% B (20–25 min); 98% B (25–30 min); 90–5% B (30–35 min). Continuum mass spectra data were acquired on an ESI-LTQ-Orbitrap-XL MS (Thermo Scientific, Bremen, Germany; ESI, electrospray ionization) with spray voltage of 2.00 kV and heated capillary temperature set at 175°C in the data-dependent mode of acquisition to automatically switch between Orbitrap-MS and LTQ-MS/MS acquisition. The six most intense precursor ions were sequentially isolated for fragmentation in the LTQ with CID, and the normalized collision energy was set to 35% with activation time of 30 ms. Activation Q was 0.25. Dynamic exclusion settings were repeat counts 2, repeat duration 30 s and exclusion duration 90 s. Survey full scan MS spectra (from m/z 350 to 1800) were acquired in the Orbitrap.

Three independent experiments were carried out. The proteins identified were similar in the three experiments.

### Data Processing and Protein Annotation

Raw files were acquired with Xcalibur 2.1.0 and converted into MGF format by Thermo Proteome Discoverer software v. 1.1.0.263 (Thermo Scientific, CA). The exported MGF files were searched against the local database downloaded from UniProtKB (Taxonomy: Viridiplantae, containing 36,020 sequences) with the MASCOT software (version 2.3, installed on a local server). The search criteria were as follows: enzyme, trypsin; fixed modification, carbamidomethyl (cysteine); variable modification, oxidation (methionine); peptide tolerance, 10 ppm; fragment mass tolerance, ±0.6 Da; peptide charge state, 2+, 3+; instrument profile, ESI-Trap; and one max missed cleavage. Hits were considered high confidence if at least three peptides were matched with ion scores >25 or proteins were identified by one or two peptides with score ≥40.

### Real-Time Quantitative PCR

**FIGURE 1:**
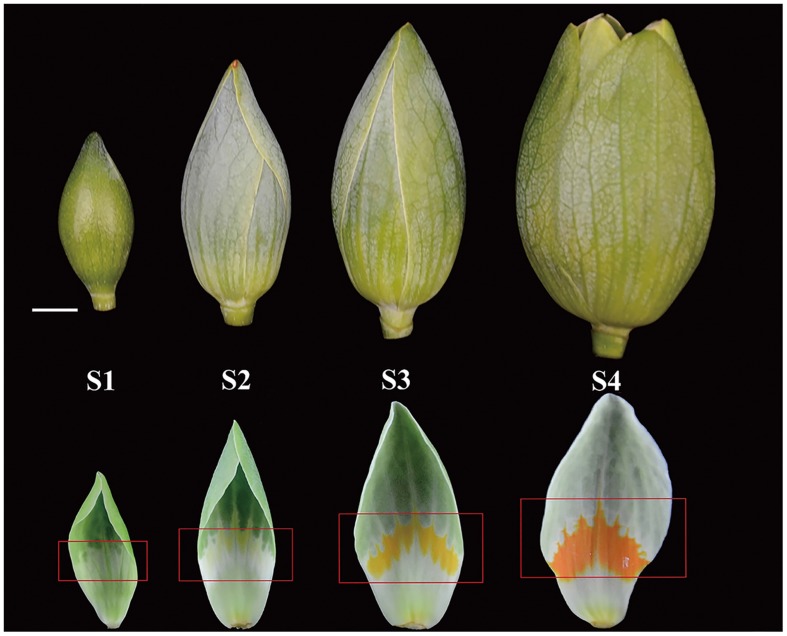
**Developmental stages of *Liriodendron tulipifera* flower buds and nectaries.** (S1) Ten days before nectar secretion, the nectary area was light green; (S2) 3 days before secreting nectar, nectary area beginning to yellow; (S3) flowers beginning to intumesce and secrete nectar, nectary area fully yellow; (S4) 20 h after stage 3, nectary area was bright orange. Red rectangles delineate the nectary area on the petals. The white bar is 1 cm.

Leaf buds, shoot and leaves were collected with the nectar, as well as petals, bract, stamen, and stigma of highly secreting flowers. The different stages of nectary development were designated S1–S4 (**Figure 1**). Stage 1 (S1): 10 days before nectar secretion, the nectary area was light green. Stage 2 (S2): 3 days before secreting nectar, nectary area beginning to yellow. Stage 3 (S3): flowers beginning to intumesce and secrete nectar, nectary area fully yellow. Stage 4 (S4): 20 h after stage 3, nectary area was bright orange. Total RNA of all samples was extracted using the Total RNA Purification kit (Norgen, Canada). Quality and concentration of isolated RNA were assessed with a Nanodrop 2000 (Thermo Fisher Scientific, USA) and agarose gel electrophoresis.

cDNA was synthesized from total RNA using the SuperScript III First-Strand Synthesis System for RT-PCR (Invitrogen, USA). Specific primers were designed according to the open reading frames in the scaffolds (Supplementary Table S1) using MacVector 11.1.0 software (MacVector, USA). The primers are shown in Supplementary Table S2. According to preliminary experiments, the optimal amount of cDNA and the number of PCR cycles corresponding to the exponential phase of the reaction were determined. Constitutively expressed 18S rRNA was amplified (primers also shown in Supplementary Table S2) as a loading control.

To investigate the relative expression of genes in different organs and stages, real-time PCR were performed using the LightCycler 480 II Real-Time PCR System with the LightCycler 480 SYBR Green I Master mix (Roche, Switzerland) and the following program: 5 min at 95°C (1 cycle); 10 s at 95°C, 15 s at 60°C and 20 s at 72°C (45 cycles). Semi-quantitative RT PCR was then performed according to the protocol in the KOD FX Polymerase manual (Toyobo, Japan).

The experiments shown are representative of independent experiments with triplicate assays.

### Bioinformatic Analysis

To facilitate analysis of the functions of *L. tulipifera* nectar proteins and also to provide a resource for the study of other nectarines, we used Blast2GO assignments to annotate the nectar proteins with gene ontology (GO) terms. All of the annotated proteins were submitted to GO analysis using Blast2GO 3.0.9 software (Conesa et al., 2005) with default parameters. The analysis was performed by searching (BLASTp) the translated protein sequences from the open reading frames in the scaffolds (unpublished data) against the SWISS-PROT database using the public NCBI BLAST service. Only the statistically significant alignments (<1.0 E^–5^) were considered.

To better understand the functions and interactions of the identified *L. tulipifera* nectar proteins, a protein–protein interaction network analysis was performed with the online analysis tool STRING^1^ (version 10) with a confidence level of 4. Because the protein annotation was based on different organisms in the SWISS-PROT Viridiplantae database, all identified proteins were searched against the *Arabidopsis thaliana* protein database to obtain annotated protein entries for the network analysis.

Protein hydrophobicity was analyzed with ProtScale, one of the ExPASy online tools, with the Kyte & Doolittle hydrophobicity scale for amino acids. The BioEdit software version 7.0.9.0 was used to predict amino acid frequencies. All parameters were the default.

To examine the phylogenetic position of *L. tulipifera* REF sequence, we selected reported and predicted REF protein sequences of 15 species belonging to 15 different families representing order Eubryales to Brassicales. The multiple sequence alignment was trimmed using trimAL version 1.3 (Capella-Gutiérrez et al., 2009) and the phylogenetic analysis was performed with the MEGA 6 software (Tamura et al., 2013). The protein sequences were used to construct a maximum-likelihood phylogenetic tree (**Figure 6D**) and *Physcomitrella patens* was set as the outgroup. Initial trees for the heuristic search were obtained by applying the neighbor-joining method to a matrix of pairwise distances estimated using an LG model. A discrete Gamma distribution was used to model evolutionary rate differences among sites (*G* = 5, parameter = 1.4675). The tree is drawn to scale, with branch lengths measured in the number of substitutions per site. The 15 amino acid sequences were downloaded from the NCBI Viridiplantae database (Supplementary Table S3).

## Results and Discussion

### *L. tulipifera* General Floral Nectar Traits and Nectary Morphology

*Liriodendron tulipifera* nectar was first secreted when the flower buds began to intumesce (S3, **Figure 1**). In this stage, the anthers was still not fully mature but the gynoecium had been mature for fertilization. A lot of yellow stripes first occurred in the nectary area. Then the secreted nectar volume reached the maximum in the following 24 h (about 2∼4 h later after S4). Meanwhile, the petals began to unfold, while the anthers first became mature and the nectary area became orange–yellow to attract pollinators in the morning. This early secretion strategy would attract multiple insects or other organisms indiscriminately and provide ample opportunity for pollination. This may be a evidence that *L. tulipifera* is an entomophilous plant. Nearly opened flowers had accumulated ∼1600–2100 μl of raw nectar per flower (*n* = 20) before pollinators visit. Because of its extraordinary nectar-producing ability and wide distribution in North America, *L. tulipifera* is valued as a nectar source for honey production and as a source of wildlife food (Liang et al., 2011). *L. tulipifera* floral nectar was acidic, with pH of 4.8 ± 0.15 (mean ± SD, *n* = 15). The mean total protein content in pooled nectar samples was 130 ± 28 μg ml^–1^ (mean ± SD, *n* = 9) and exceeded the mean value of 100 μg ml^–1^ in floral nectar of most other species (Nicolson and Thornburg, 2007b).

**FIGURE 2:**
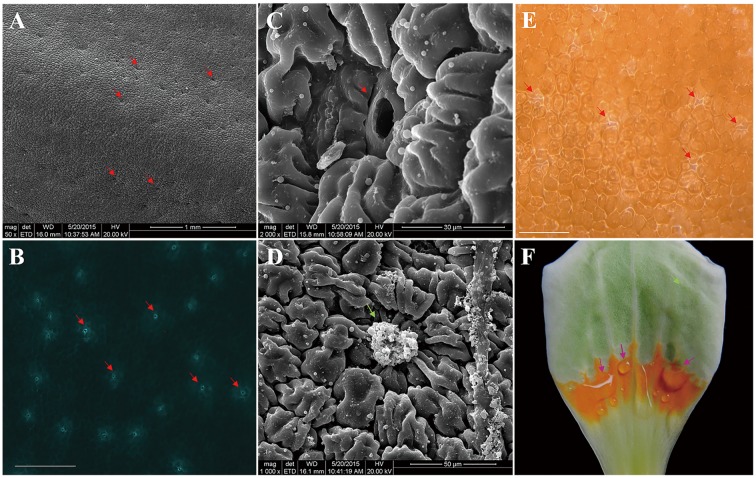
**Photographs of *L. tulipifera* nectary. (A)** Glabrous internal surface of a petal showing the secretory stomata (red arrows). Magnification is 50×. **(B)** Autofluorescence image of Secretory stomata in GFP (red arrows). Magnification is 100×. Bar is 100 μm. **(C)** Higher magnification image of a secretory stoma of the nectary. Magnification is 2000×. **(D)** Higher magnification of a secretory stoma occluded with secretory material. Magnification is 1000×. **(E)** Secreting *L. tulipifera* nectar stomata (red arrows). Bar is 100 μm. **(F)** Isolated petal showing the orange ring and raw nectar (pink arrows). Bar is 1 cm.

In stage S3, *L. tulipifera* nectar was secreted from the individual nectarostomata (**Figures 2B,E**), then formed liquid nectar drops onto the petal internal surface, where there was a yellow–orange fleshy ring (**Figure 2F**). In stage S4, the liquid nectar drops formed even bigger drops and flowed down to the base of petals. To further study the nectary structure, petals were examined by SEM. We observed that *L. tulipifera* nectar was exuded through numerous modified, sunken stomata in the epidermis of the lower glabrous part of the petals, which are made up of guard and epidermal cells (**Figure 2C**). The outer cuticular layer cells were more evident than guard cells before secretion, and they gradually collapsed after secretion (**Figures 2A,D**).

Flowers can attract pollinating insects in several ways: they can offer beneficial shelter, they may have specialized color, odor, or pheromonal attractants or they may offer floral reward (Faegri and Van der Pijl, 1971; Miller, 1883). In fact, most flower, including those of *L. tulipifera*, rely primarily on the last category (Lundgren, 2009). *L. tulipifera* nectaries secrete thousands of μl of nectar per flower, whereas nectaries in most species generally produce <10 μl (Pacini et al., 2003). Furthermore, like the brightly colored flowers of others species, the colorful nectary of *L. tulipifera* attracts insects (Kevan, 1972). In conclusion, incorporation of these multiple strategies to attract insects can improve the fecundity of an entomophilous plant.

Nectary structure and position can differ among flowers to the point of being useful for taxonomic classification (Fahn, 1979). The nectary is typically composed of epidermis, with or without stomata, which normally mediates nectar release; parenchyma, which produces or stores substances that become dissolved in the nectar; and the vascular bundle, which conveys water or nutrients to the parenchyma (Pacini et al., 2003). Nectar secretion through stomata is the most common manner of nectar release (Bernardello, 2007; Nepi, 2007). Although *L. tulipifera* nectar is secreted through nectarostomata, the *L. tulipifera* flower nectary structure is very different than that of Brassicaceae flowers (Kram and Carter, 2009) and even that of *Magnolia stellata* in the same Magnoliaceae family (Erbar, 2014). As an apocarpous gynoecium flower, the nectary of *L. tulipifera* flowers was located on the modified orange–yellow part of petals, as in flowers of *Helleborus* and *Symphyglossum* (Vesprini et al., 1999; Stpiczynska and Davies, 2006), whereas in most species, it encircles the ovary (Brown, 1938; Zer and Fahn, 1992; Rivera, 2000; Konarska, 2010; Nocentini et al., 2012; Stpiczyñska et al., 2012; Lüttge, 2013; Stephens, 2013). Although this result was consistent with previous findings in plants in the Ranunculaceae family (Kosuge, 1994), it was different from what has been seen in *M. stellata* in the same family (Erbar and Leins, 2013). As the secreting petals produce a colorful ring, we hypothesize that the bright-colored and glabrous nectary tissues of *L. tulipifera* may be more favorable for attracting its pollinator.

### *L. tulipifera* Nectarin Annotation by Gel Electrophoresis and LC-MS/MS

**FIGURE 3:**
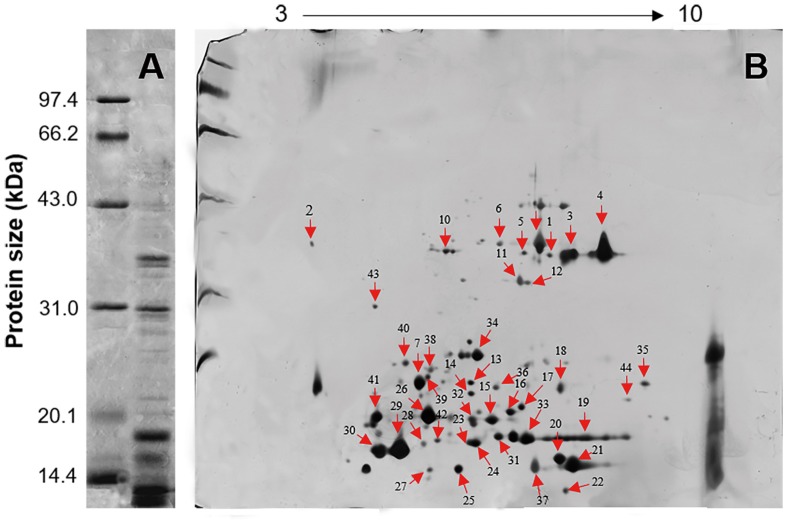
**Gel electrophoresis of *L. tulipifera* nectar proteins. (A)** 1D gel electrophoresis of *L. tulipifera* nectar proteins. Lane 1, reference proteins; lane 2, 10 μg of *L. tulipifera* nectar protein. **(B)** Identification of *L. tulipifera* nectar proteins (numbered arrows) separated by 2D gel electrophoresis using nanoLC-MS/MS analysis. Reference proteins were the same as in A and 120 μg of nectar protein was loaded. The protein identifications are listed in **Table 1.**

Five distinct main bands, ranging in size from 10 to 41 kDa, were yielded by 1-D SDS-PAGE and visualized by Coomassie Brilliant Blue R-250 staining under reducing conditions (**Figure 3A**). The 2-D gel electrophoresis showed that most of the *L. tulipifera* nectarines were acidic proteins ranging in molecular mass from 10 to 40 kDa, consistent with the patterns observed in the 1-D gel electrophoresis (**Figure 3B**).

Individual *L. tulipifera* nectar proteins were further separated through 2-D gel electrophoresis, and 42 proteins were successfully annotated (**Table 1**) by searching against the SWISS-PROT database downloaded from UniProtKB. The proteins between 20 and 30 kDa were annotated as binding proteins, ribosylation factor, amino acid transferase and reductase (**Figure 3B**; **Table 1**). Those between 14 and 20 kDa were annotated as small ubiquitin-related modifier and carbonic anhydrase (**Figure 3B**; **Table 1**). The most abundant proteins below 14 kDa were ubiquitin-related proteins, REF, glutaredoxin and profilin (**Figure 3B**; **Table 1**). Interestingly, ubiquitin-related proteins and REF have not been observed in flower nectar of other plants, and no nectarin (NEC)-like proteins were found in *L. tulipifera* nectar (Carter et al., 1999; Ge et al., 2000; Carter and Thornburg, 2004b,c; Saglar Naqvi et al., 2005). However, carbonic anhydrase was detected in our survey and NEC3 in ornamental tobacco plants has been demonstrated to have the same carbonic anhydrase activity (Carter and Thornburg, 2004b). Therefore, some *L. tulipifera* nectar proteins may have analogous functions with known nectarines, but more information is needed to characterize their homology.

**Table 1:** Floral nectar proteins of *Liriodendron tulipifera* Linn. (South Carolina accession) annotated by LC-MS/MS after 2-D gel electrophoresis.

Spot	Accession	Protein name	Score	Coverage %	MW/pI^∗^	Hit Peptides	Species
1	A2Y7R5	GTP-binding nuclear protein Ran-2	225	19	25.36/6.66	LVIVGDGGTGK HLTGEFEK VCENIPIVLCGNK NLQYYEISAK	*Oryza sativa* Indica Group
2	P15252	Rubber elongation factor protein	160	34	14.71/5.04	SGPLQPGVDIIEGPVK NVAVPLYNR DASIQVVSAIR SLASSLPGQTK	*Hevea brasiliensis*
3	A2Y7R5	GTP-binding nuclear protein Ran-2	162	21	25.36/6.66	HLTGEFEK LVIVGDGGTGK NLQYYEISAK VCENIPIVLCGNK	*Oryza sativa* Indica Group
4	P15252	Rubber elongation factor protein	244	27	14.71/5.04	SGPLQPGVDIIEGPVK DASIQVVSAIR SLASSLPGQTK	*Hevea brasiliensis*
5	Q43209	Protein-L-isoaspartate *O*-methyltransferase	451	7	24.81/4.90	VAEVMETIDR YVPLTSR	*Triticum aestivum*
6	P11143	Heat shock 70 kDa protein	814	18	70.87/5.22	VEIIANDQGNR TTPSYVAFTDTER QFAAEEISSMVLIK EIAEAYLGSTIK NAVVTVPAYFNDSQR DAGVIAGLNVMR IINEPTAAAIAYGLDK ATAGDTHLGGEDFDNR MVNHFVQEFK	*Zea mays*
7	O82089	Copper transport protein CCH	255	12	13.08/4.91	MEGVESFDIDIK MEGVESFDIDIKEQK	*Arabidopsis thaliana*
10	Q9FRL8	Glutathione *S*-transferase DHAR2	115	9	23.51/5.79	VLLTLEEK NWSVPESLTSVR	*Arabidopsis thaliana*
11	Q0JNR2	Cysteine proteinase inhibitor 12	322	3	27.25/6.07	ENALLEFVR	*Oryza sativa* Japonica Group
12	Q9SVD7	Ubiquitin-conjugating enzyme E2 variant 1D	264	34	16.69/6.20	TLGSGGSSVVVPR LLEELER GIGDGTVSYGMDDGDDIYMR KLVQPPEGTCF	*Arabidopsis thaliana*
13	P49310	Glycine-rich RNA-binding protein GRP1A	634	38	16.06/5.21	ASPDVEYR CFVGGLAWATDDR DAIEGMNGQDLDGR SITVNEAQSR SGGGGGYGGGGGGYGGGGR	*Sinapis alba*
14	A2XKU9	Costars family protein	173	27	9.63/5.78	VTFGVIFNDDR CANIFEALVGTLR	*Oryza sativa* Indica Group
15	P29449	Thioredoxin H-type 1	2221	18	14.12/5.62	KLVVVDFTASWCGPCR LVVVDFTASWCGPCR VDVDELK	*Nicotiana tabacum*
16	P15252	Rubber elongation factor protein	378	52	14.71/5.04	SGPLQPGVDIIEGPVK NVAVPLYNR FSYIPNGALK FVDSTVVASVTIIDR DASIQVVSAIR SLASSLPGQTK	*Hevea brasiliensis*
	P29449	Thioredoxin H-type 1	217	18	14.12/5.62	VDVDELK LVVVDFTASWCGPCR KLVVVDFTASWCGPCR	*Nicotiana tabacum*
17	Q84JT6	Peptide methionine sulfoxide reductase B9	128	12	15.75/6.81	AILSPEQFR HCVNSVSLK	*Arabidopsis thaliana*
18	P35135	Ubiquitin-conjugating enzyme E2-17 kDa	237	47	16.68/7.71	ELKDLQK VFHPNINSNGSICLDILK EQWSPALTISK VLLSICSLLTDPNPDDPLVPEIAHMYK AKYETTAR	*Solanum lycopersicum*
	Q03250	Glycine-rich RNA-binding protein 7	633	21	16.94/5.85	SITVNEAQSR CFVGGLAWATDDR DAIEGMNGQDLDGR	*Arabidopsis thaliana*
	Q9FVI1	Actin-depolymerizing factor 2	168	13	16.67/5.78	MIYASSK IFFIAWSPDTAR	*Petunia* × *hybrida*
19	Q94A97	Ubiquitin-conjugating enzyme E2 35	223	27	17.24/6.74	LLSEPAPGISASPSEDNMR LELFLPEEYPMAAPK IYHPNIDK	*Arabidopsis thaliana*
	P55142	Glutaredoxin-C6	61	30	11.94/5.77	TVPNVFINGK AIELDGESDGSELQSALAEWTGQR	*Oryza sativa* Japonica Group
20	P69310	Ubiquitin	281	61	8.52/6.56	TITLEVESSDTIDNVK IQDKEGIPPDQQR EGIPPDQQR TLADYNIQK ESTLHLVLR	*Avena sativa*
21	P0CH10	Ubiquitin-60S ribosomal protein L40	2014	54	14.92/9.94	EGIPPDQQR TLADYNIQK ESTLHLVLR MQIFVKTLTGK LIFAGKQLEDGR IQDKEGIPPDQQR TITLEVESSDTIENVK TLADYNIQKESTLHLVLR	*Chlamydomonas reinhardtii*
22	P84718	Putative oxygen-evolving enhancer protein 1	141	24	12.27/4.35	VINTWADIINR GGSTGYDNAVALPAGGR	*Pinus strobus*
23	P69310	Ubiquitin	184	61	8.52/6.56	TITLEVESSDTIDNVK IQDKEGIPPDQQR TLADYNIQK ESTLHLVLR	*Avena sativa*
	P15252	Rubber elongation factor protein	318	44	14.71/5.04	NVAVPLYNR SLASSLPGQTK FSYIPNGALK SGPLQPGVDIIEGPVK FVDSTVVASVTIIDR	*Hevea brasiliensis*
24	P29449	Thioredoxin H-type 1	285	18	14.12/5.62	KLVVVDFTASWCGPCR LVVVDFTASWCGPCR VDVDELK	*Nicotiana tabacum*
25	P0C030	Ubiquitin-NEDD8-like protein RUB1	1629	62	17.12/5.77	TITLEVESSDTIDNVK IQDKEGIPPDQQR EGIPPDQQR TLTGKEIEIDIEPTDTIDR EIEIDIEPTDTIDR VEEKEGIPPVQQR EGIPPVQQR DYNIEGGSVLHLVLALR	*Oryza sativa* Japonica Group
	A2XKU9	Costars family protein	181	27	9.63/5.78	VTFGVIFNDDR CANIFEALVGTLR	*Oryza sativa* Indica Group
26	P29449	Thioredoxin H-type 1	1314	25	14.12/5.62	VDVDELK EVDRVVGAK LVVVDFTASWCGPCR KLVVVDFTASWCGPCR	*Nicotiana tabacum*
	Q03250	Glycine-rich RNA-binding protein 7	675	21	16.94/5.85	CFVGGLAWATDDR DAIEGMNGQDLDGR SITVNEAQSR	*Arabidopsis thaliana*
	B4YYA9	Costars family protein ST45-2	291	26	10.19/5.63	VTFGVLFNDDR CANIFEALVGTLR	*Eutrema halophilum*
27	P0C030	Ubiquitin-NEDD8-like protein RUB1	265	42	17.12/5.77	TITLEVESSDTIDNVK TLADYNIQK EIEIDIEPTDTIDR EGIPPVQQR DYNIEGGSVLHLVLALR	*Oryza sativa* Japonica Group
28	P15252	Rubber elongation factor protein	215	41	14.71/5.04	SGPLQPGVDIIEGPVK NVAVPLYNR FSYIPNGALK DASIQVVSAIR SLASSLPGQTK	*Hevea brasiliensis*
29	P15252	Rubber elongation factor protein	530	52	14.71/5.04	SGPLQPGVDIIEGPVK NVAVPLYNR FSYIPNGALK FVDSTVVASVTIIDR DASIQVVSAIR	*Hevea brasiliensis*
30	P15252	Rubber elongation factor protein	283	41	14.71/5.04	SGPLQPGVDIIEGPVK NVAVPLYNR FSYIPNGALK DASIQVVSAIR SLASSLPGQTK	*Hevea brasiliensis*
31	A4KA43	Profilin-6	120	9	14.19/4.90	YMVIQGEPGVVIR	*Corylus avellana*
	P15252	Rubber elongation factor protein	111	44	14.71/5.04	NVAVPLYNR SLASSLPGQTK DASIQVVSAIR SGPLQPGVDIIEGPVK FVDSTVVASVTIIDR	*Hevea brasiliensis*
32	A2YIW7	Thioredoxin H-type	112	5	13.32/5.16	VDVDELK	*Oryza sativa* Indica Group
33	Q9C996	GDSL esterase	109	2	40.65/8.38	CFGKMNVMAK	*Arabidopsis thaliana*
34	P0DH9	ADP-ribosylation factor 2-B	124	19	20.64/6.43	ILMVGLDAAGK DAVLLVFANK QDLPNAMNAAEITDK	*Glycine soja*
35	O49886	Peptidyl-prolyl *cis*-trans isomerase	98	12	18.51/8.36	TAENFR FADENFIK FADENFIKK TEWLDGK	*Lupinus luteus*
	P49310	Glycine-rich RNA-binding protein GRP1A	204	33	16.06/5.21	SITVNEAQSR CFVGGLAWATDDR DAIEGMNGQDLDGR	*Sinapis alba*
36	Q9FVI1	Actin-depolymerizing factor 2	2168	13	16.67/5.78	MIYASSK IFFIAWSPDTAR	*Petunia* × *hybrida*
37	P0CG86	Ubiquitin-40S ribosomal protein S27a	158	28	17.87/9.83	MQIFVK IQDKEGIPPDQQR TLADYNIQK ESTLHLVLR VDDATGKVTR	*Hordeum vulgare*
38	P15252	Rubber elongation factor protein	439	52	14.71/5.04	NVAVPLYNR SLASSLPGQTK FSYIPNGALK DASIQVVSAIR FVDSTVVASVTIIDR	*Hevea brasiliensis*
	Q9FVI1	Actin-depolymerizing factor 2	765	18	16.67/5.78	MIYASSK QKEVVVEK IFFIAWSPDTAR	*Petunia* × *hybrida*
39	Q03250	Glycine-rich RNA-binding protein 7	326	21	16.94/5.85	SITVNEAQSR CFVGGLAWATDDR DAIEGMNGQDLDGR	*Arabidopsis thaliana*
	132270	Rubber elongation factor protein	338	52	14.71/5.04	SGPLQPGVDIIEGPVK NVAVPLYNR FSYIPNGALK FVDSTVVASVTIIDR DASIQVVSAIR SLASSLPGQTK	*Hevea brasiliensis*
40	Q9FLP6	Small ubiquitin-related modifier 2	439	35	11.76/5.35	LMNAYCDR GQDGNEVFFR KLMNAYCDR VKGQDGNEVFFR QSVDFNSIAFLFDGR	*Arabidopsis thaliana*
	Q9FVI1	Actin-depolymerizing factor 2	188	13	16.67/5.78	MIYASSK IFFIAWSPDTAR	*Petunia* × *hybrida*
41	Q9SNW5	Profilin-3	600	16	14.27/4.73	KGSGGVTIK YMVIQGEPGAVIR	*Lilium longiflorum*
42	A4KA43	Profilin-6	169	9	14.19/4.90	YMVIQGEPGVVIR	*Corylus avellana*
	P15252	Rubber elongation factor protein	167	26	14.71/5.04	SGPLQPGVDIIEGPVK NVAVPLYNR SLASSLPGQTK	*Hevea brasiliensis*
43	Q9ZSW9	Translationally-controlled tumor protein homolog	673	27	19.08/4.50	QFVTYMK VVDIVDTFR LQEQPAFDK MLVYQDLLTGDELLSDSFPYK	*Hevea brasiliensis*
	Q0JNS6	Calmodulin-1	181	37	16.88/4.11	ELGTVMR HVMTNLGEK LTDEEVDEMIR DTDSEEELKEAFR VFDKDQNGFISAAELR	*Oryza sativa* Japonica Group
44	Q5Z9Z3	Thioredoxin-like protein Clot	219	7	15.34/4.88	LTGVPTLIR FRLTGVPTLIR	*Oryza sativa* Japonica Group

^∗^*The numerical value of Mw and pI just represent the accession, not the experimental value.*

Although it has been known that nectar contains proteins for some time, few nectarines have been characterized in detail (Peumans et al., 1997; Carter and Thornburg, 2000, 2004b,c; Thornburg et al., 2003; Kram et al., 2008; Harper et al., 2010; Hillwig et al., 2010, 2011; Nepi et al., 2011; Zha et al., 2013). During our proteomics survey, we detected isomerase, transferase, carbonic anhydrase, short-chain dehydrogenase reductase, ATPase, diphosphate kinase, and GDSL esterase in *L. tulipifera* floral nectar. One of GDSL esterase/lipases identified in our survey (spot 33) has been reported to have antimicrobial activities in both *Jacaranda mimosifolia* (Kram et al., 2008) and *Arabidopsis* (Oh et al., 2005) nectar. A diversity of defense proteins, like NADPH oxidase, endochitinase, β-1, 3-glucanases and xylosidase have been shown to defense against microorganisms in floral and extrafloral nectar (Carter and Thornburg, 2000, 2004a; Saglar Naqvi et al., 2005; Carter et al., 2007; Kram et al., 2008; González-Teuber et al., 2009, 2010; Hillwig et al., 2011; Nepi et al., 2011). Thus, we speculate that the enzymes identified in *L. tulipifera* nectar may also play an important role in the interaction of nectar with the biotic environment. The functions of other enzymes in the nectar are still unclear and may be explored in future studies.

The ubiquitin-related proteins are involved in many biological processes in almost all organisms. The presence of these proteins in secreted nectar may indicate contamination of the secreted floral nectar with cellular proteins arising from natural cellular degradation occurring during the nectar secretion process. This cellular degradation has previously been observed in the ornamental tobacco nectary (Carter et al., 2007).

### Classification of *L. tulipifera* Nectar Proteins

**FIGURE 4:**
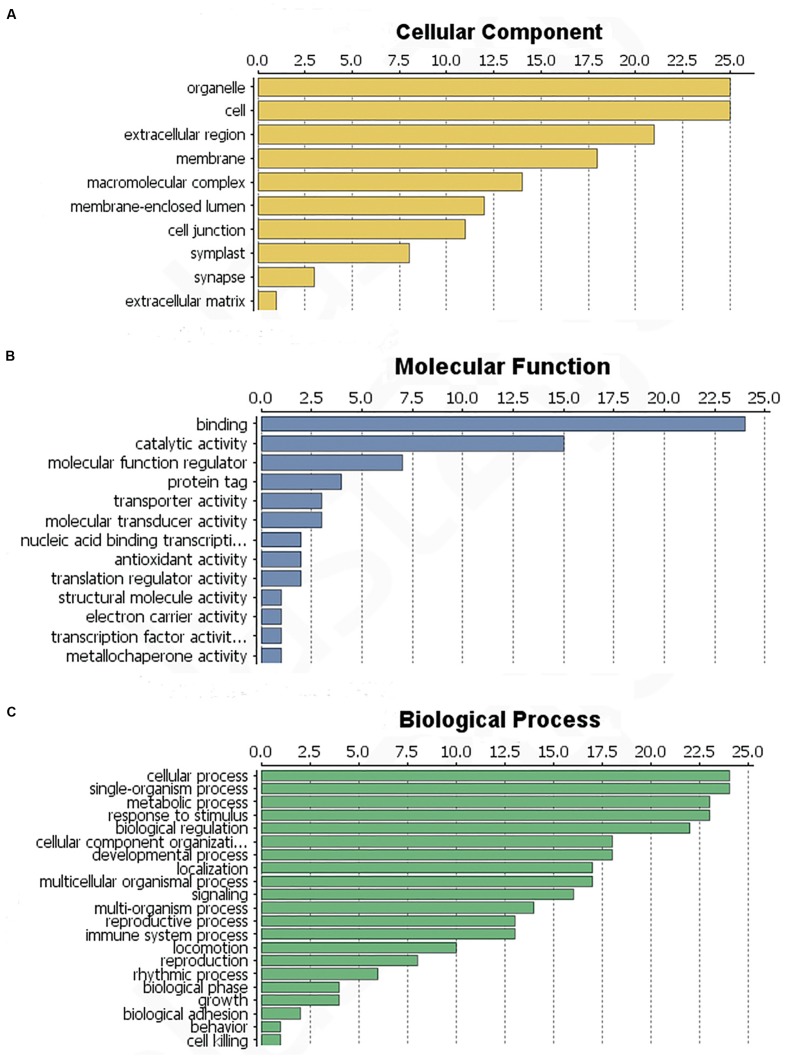
**Functional classification of the identified *L. tulipifera* floral nectar proteins. (A)** Cellular component profile. **(B)** Molecular function profile. **(C)** Biological process distribution. Numbers on the x-axes indicate the number of proteins.

The identified proteins were categorized according to the three main GO categories: cellular component, molecular function, and biological process. In terms of cellular component, the largest group of proteins corresponded to the cell and organelle subcategories, followed by extracellular region, membranes, macromolecular complexes and the membrane-enclosed lumen (**Figure 4A**). In molecular function category of the identified proteins, the most prominent was binding, catalytic activity and molecular function regulation. Antioxidant activity was also found, but in a low component (**Figure 4B**). For biological process category, single-organism processes, response to stimulus, cellular processes and metabolic process were top terms (**Figure 4C**). In summary, most of the identified proteins were involved in catalytic activity, binding and antioxidant activity, response to stimulus and immune system processes.

**FIGURE 5:**
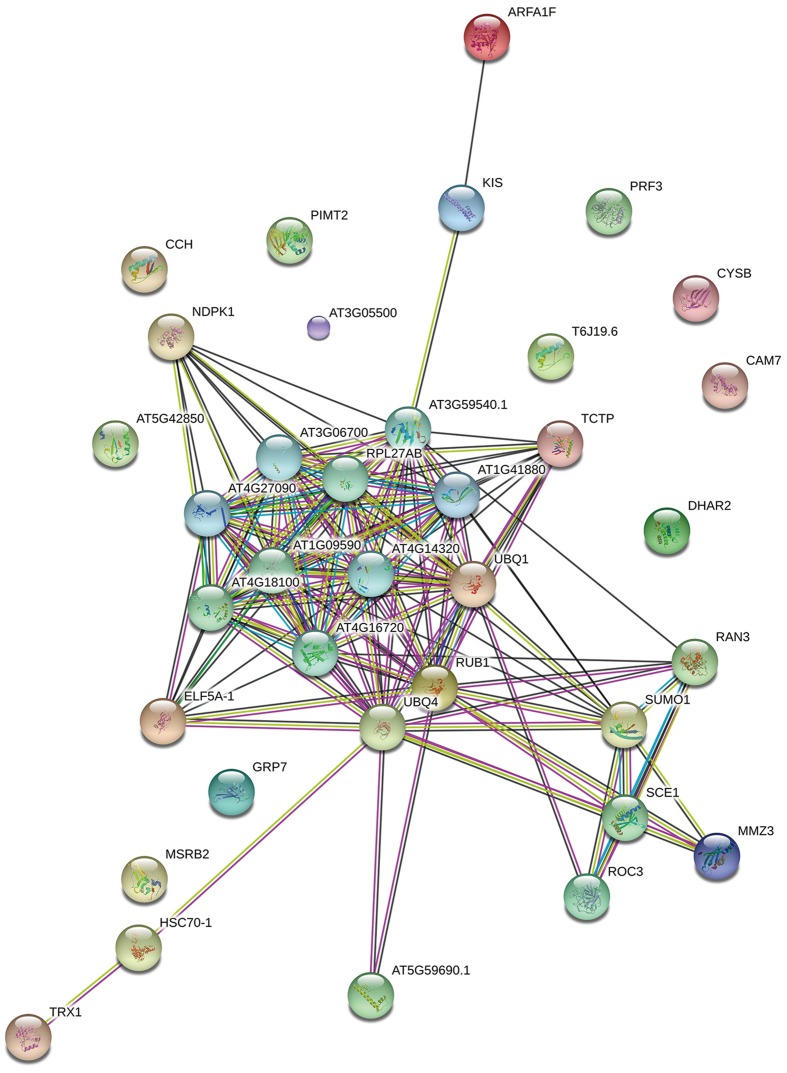
**Functional networks of the identified *L. tulipifera* floral nectar proteins.** Different line colors represent the types of evidence used in predicting the associations: gene fusion (red), neighborhood (green), co-occurrence across genomes (blue), co-expression (black), experimental (purple), association in curated databases (light blue), text mining (yellow), homology (light purple).

To determine the function of proteins involved in the various biological processes and the protein–protein interactions, the identified protein sequences were submitted to STRING online. The protein–protein interactions are shown in **Figure 5.** Many ubiquitination-related proteins and kinases were involved in the predicted interactions. Ubiquitination is an important post-translational protein modification and regulates a wide range of cellular processes, including responses to hormones, light, sucrose, development signals, and pathogens (Ellis et al., 2002). Previous studies revealed that ubiquitination may also play an important role in plant defense against pathogens. (Xie et al., 1998; Kim and Delaney, 2002; Devoto et al., 2003; Lee et al., 2007). This suggests that *L. tulipifera* flower nectar may have defense functions. This new protein database will be a valuable resource for further studies on *L. tulipifera* flower nectar proteins.

### Sequence and Expression Analysis of *L. tulipifera* REF

Among the proteins that were identified by our approach, we found *L. tulipifera* REF (spot 4), which had not been detected in previous nectar research. REF, a member of the REF/SRPP-like protein family, is the major allergen in latex (Czuppon et al., 1993), and it has also been shown to be involved in resistance mechanisms (Ko et al., 2003).

**FIGURE 6:**
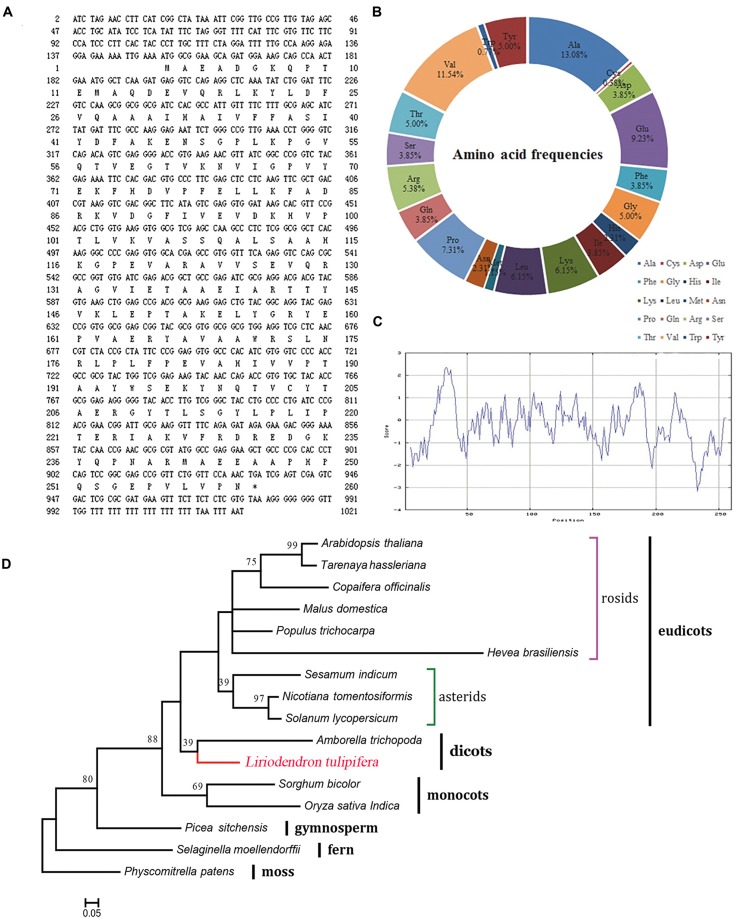
**Characterization of the REF protein from *L. tulipifera*. (A)** The nucleotide and predicted amino acid sequences. The asterisk marks the stop codon (TGA). **(B)** The amino acid frequencies. **(C)** ExPasy ProtScale hydrophobicity analysis with the Kyte & Doolittle scale and window size of 9. Positive values indicate hydrophobicity and negative values indicate hydrophilicity. **(D)** Maximum-likelihood phylogenetic tree of the homologous amino acid sequences. The scale bar indicates the branch length that corresponds to 0.05 substitutions per site. The red line shows *L. tulipifera*. The species and accession numbers are listed in Supplementary Table S3. Numbers below each node are bootstrap support values.

The open reading frame is 1021 bp in length, encoding a putative 260-amino acid protein (**Figure 6A**). The theoretical isoelectric point is 5.93 and the molecular weight is 28.82 kDa. To verify the conserved domain we submitted the protein sequence to the SMART server and aligned with *Hevea brasiliensis* REF sequence (Hev b1, P15252). The protein sequence contains a 216-aa conserved domain and is highly consistent with the *H. brasiliensis* sequence (Supplementary Figure S1). The frequencies of amino acids were deduced with the BioEdit software. Notably, alanine and cysteine were the most (13.08%) and least (0.38%) frequently coded amino acids (**Figure 6B**), respectively, and the peptide contained all 20 amino acids. The protein was predicted to be hydrophilic, with an overall average hydrophobicity score of -0.238 (**Figure 6C**).

Phylogenetically, *L. tulipifera* REF fell into the clade of dicots between monocots and eudicots, and it was closely related to *Amborella trichopoda*. This phylogenetic topology was congruent with previous phylogenetic analyses (Berthelot et al., 2014b); further supporting that *L. tulipifera* REF is a REF protein.

**FIGURE 7:**
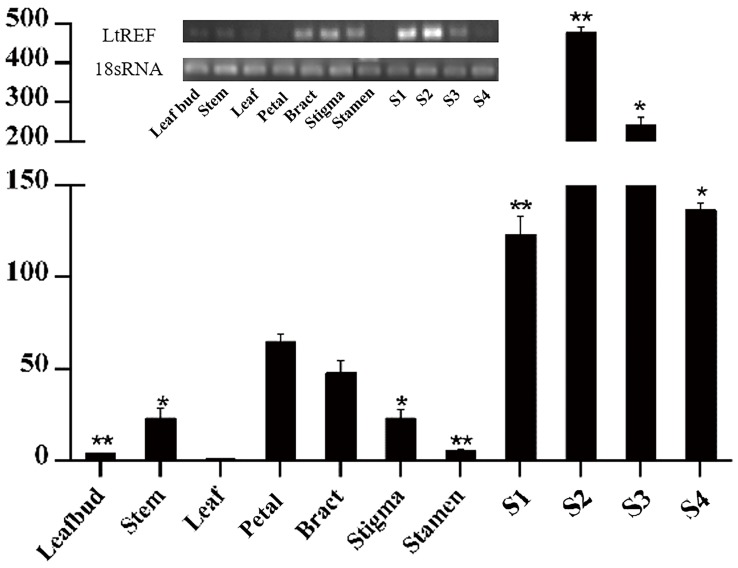
**Quantitation of expression of the REF gene in different tissues of *L. tulipifera*.** S1–S4 correspond to the nectary tissue developmental stages in **Figure 1** (^∗∗^*P* < 0.01, ^∗^*P* < 0.05).

To further analyze the *L. tulipifera* REF, we performed real-time PCR to detect expression of the *L. tulipifera* REF gene in different tissues and, in particular, in different stages of nectary development. REF’s expression was scarcely detectable in leaves, leaf bud, and stem, and was higher in petals and bracts. By contrast, the expression was dramatically higher in the nectary. To be more specific, the expression was the highest in S2 and steadily decreased in S3 and S4 (**Figure 7**). In summary, the expression in reproductive organs was higher than that in vegetative organs. As a whole, the tendencies observed in the real-time PCR were consistent with the semi-quantitative RT PCR results (**Figure 7**). The increased expression of *L. tulipifera* REF in S2 may be in preparation for protein secretion in S3.

We found that *L. tulipifera* REF, like MuSI in sweet potato (Seo et al., 2010; Kim et al., 2011), contains a REF domain and is highly similar to *H. brasiliensis* REF. REF is also able to interact with the membranes of yeasts and erythrocytes, leading to their agglutination (Berthelot et al., 2014a). However, antifungal or antimicrobial activities of REF have not been clearly demonstrated (Kanokwiroon, 2007). It is thus possible that REF has antimicrobial or other activities, and, given the homology, *L. tulipifera* REF may play a defensive role in *L. tulipifera* nectar.

## Conclusion

In our investigation, we first characterized the nectary structure of *L. tulipifera* by morphological observation. The *L. tulipifera* nectar was secreted from modificatory stoma as most other species. Nonetheless, the nectary of *L. tulipifera* was positioned in a more colorful and accessible area on the petals and secreted considerably more nectar, which was distinctly different from other genus in Magnoliaceae. These differences were vitally advantageous to attract insects for pollination.

More importantly, we applied proteomic and bioinformatic approaches to obtain a proteomic description of *L. tulipifera* nectar. Among the 42 identified proteins in the nectar of *L. tulipifera*, most of them are involved in catalytic activity, antioxidant activity, response to stimulus, biological regulation and immune system processes. In addition, a REF protein was also detected in nectar for the first time. Further bioinformation and expression analysis suggested that *L. tulipifera* REF have allergic characteristic and may play a defensive role against microorganisms in nectar. This research provides valuable information both on nectary structure and proteins in nectar, but further studies are necessary to fully elucidate the functions of all nectar proteins.

## Author Contributions

JC and JS designed the experiment, prepared samples and drafted the manuscript. YZ, ML, FZ, HZ, and LY performed the experiment. YL and GW contributed to the reagents and performed the experiment. YZ analyzed, interpreted the LC-MS/MS data, and drafted the manuscript. All authors contributed to and approved the final manuscript.

## Conflict of Interest Statement

The authors declare that the research was conducted in the absence of any commercial or financial relationships that could be construed as a potential conflict of interest.

## Footnotes

1 http://string-db.org/
