# Supplementary material for: Floral Nectary Morphology and Proteomic Analysis of Nectar of Liriodendron tulipifera Linn
Source: Front Plant Sci. 2016 Jun 14;7:826. doi: 10.3389/fpls.2016.00826 (PMC4905952; doi:10.3389/fpls.2016.00826)
Supplement: Supplementary file 1 [file Table_1.DOCX]

**Supplemental Table 1.**

**Sequence of LOC_Contig828_XA (*L. tulipifera* REF):**

GATCTAGAACCTTCATCGGCTATAAATTCGGTTGCCGTTGTAGAGCACCTGCATATCCTCATATTTCTAGGGTTTTCATTTCGTGTTCTTCCCATCCCTTCACTACCCTTGCTTTCTAGGATTTTTGCCAAGGAGAGGAGAAAAATTGAAAATGGCGGAAGCAGATGGAAAGCAGCCAACTGAAATGGCTCAAGATGAGGTCCAGAGGCTCAAATATCTGGATTTCGTCCAAGCGGCGGCGATCCACGCCATTGTTTTCTTTGCGAGCATCTATGATTTCGCCAAGGAGAATTCTGGGCCGTTGAAACCTGGGGTCCAGACAGTCGAGGGGACCGTGAAGAACGTTATCGGCCCGGTCTACGAGAAATTCCACGACGTGCCCTTCGAGCTCCTCAAGTTCGCTGACCGTAAGGTCGACGGCTTCATAGTCGAGGTGGATAAGCACGTTCCGACGCTGGTGAAGGTGGCGTCGAGCCAAGCCCTCTCGGCGGCTCACAAGGGCCCCGAGGTGGCACGAGCCGTGGTTTCAGAGGTCCAGCGCGCCGGTGTGATCGAGACGGCTGCCGAGATCGCGAGGACGACGTACGTGAAGCTGGAGCCGACGGCGAAGGAGCTGTACGGCAGGTACGAGCCGGTGGCGGAGCGGTACGCGGTGGCGGCGTGGAGGTCGCTCAACCGTCTACCGCTATTCCCGGAGGTGGCCCACATCGTGGTCCCCACCGCCGCGTACTGGTCGGAGAAGTACAACCAGACCGTGTGCTACACCGCGGAGAGGGGGTACACCTTGTCGGGCTACCTGCCCCTGATCCCGACGGAACGGATTGCGAAGGTTTTCAGAGATAGAGAAGACGGGAAATACCAACCGAACGCGCGTATGGCCGAGGAAGCTGCCCCGCACCCTCAGTCCGGCGAGCCGGTTCTGGTTCCAAACTGATCGAGTCGAGTCGACTCGCGCGATGAAGTTTCTTCTCTCGTGTAAAGGGGGGGGGTTTGGTTTTTTTTTTTTTTTTTTTAATTTAATAA

**ORF of *L. tulipifera* REF**
ATGGCAGAAGCAGATGGAAAGCAGCCAACTGAAATGGCTCAAGATGAGGTCCAGAGGCTCAAATATCTGGATTTCGTCCAAGCGGCGGCGATCCACGCCATTGTTTTCTTTGCGAGCATCTATGATTTCGCCAAGGAGAATTCTGGGCCGTTGAAACCTGGGGTCCAGACAGTCGAGGGGACCGTGAAGAACGTTATCGGCCCGGTCTACGAGAAATTCCACGACGTGCCCTTCGAGCTCCTCAAGTTCGCTGACCGTAAGGTCGACGGCTTCATAGTCGAGGTGGATAAGCACGTTCCGACGCTGGTGAAGGTGGCGTCGAGCCAAGCCCTCTCGGCGGCTCACAAGGGCCCCGAGGTGGCACGAGCCGTGGTTTCAGAGGTCCAGCGCGCCGGTGTGATCGAGACGGCTGCCGAGATCGCGAGGACGACGTACGTGAAGCTGGAGCCGACGGCGAAGGAGCTGTACGGCAGGTACGAGCCGGTGGCGGAGCGGTACGCGGTGGCGGCGTGGAGGTCGCTCAACCGTCTACCGCTATTCCCGGAGGTGGCCCACATCGTGGTCCCCACCGCCGCGTACTGGTCGGAGAAGTACAACCAGACCGTGTGCTACACCGCGGAGAGGGGGTACACCTTGTCGGGCTACCTGCCCCTGATCCCGACGGAACGGATTGCGAAGGTTTTCAGAGATAGAGAAGACGGGAAATACCAACCGAACGCGCGTATGGCCGAGGAAGCTGCCCCGCACCCTCAGTCCGGCGAGCCGGTTCTGGTTCCAAACTGA

**Sequence of Lt18SrRNA2 (CK756221):**

GTTGATCCTGCCAGTAGTCATATGCTTGTCTCAAAGATTAAGCCATGCATGTGTAAGTATGAACTAATTCAGACTGTGAAACTGCGAATGGCTCATTAAATCAGTTATAGTTTGTTTGATGGTAACTGCTACTCGGATAACCGTAGTAATTCTAGAGCTAATACGTGCAACAAACCCCGACTTCTGGAAGGGATGCATTTATTAGATAAAAGGTCGACGCGGGCTCTGCCCGTCGCTCTGATGATTCATGATAACTTGACGGATCGCACGGCCCTCGTGCTGGCGACGCATCATTCAAATTTCTGCCCTATCAACTTTCGATGGTAGGATAGTGGCCTACTATGGTGGTGACGGGTGACGGAGAATTAGGGTTCGATTCCGGAGAGGGAGCCTGAGAAACGGCTACCACATCCAAGGAAGGCAGCAGGCGCGCAAATTACCCAATCCTGACACGGGGAGGTAGTGACAATAAATAACAATA
