# Supplementary figures and images for: Floral Nectary Morphology and Proteomic Analysis of Nectar of Liriodendron tulipifera Linn
Source: Front Plant Sci. 2016 Jun 14;7:826. doi: 10.3389/fpls.2016.00826 (PMC4905952; doi:10.3389/fpls.2016.00826)

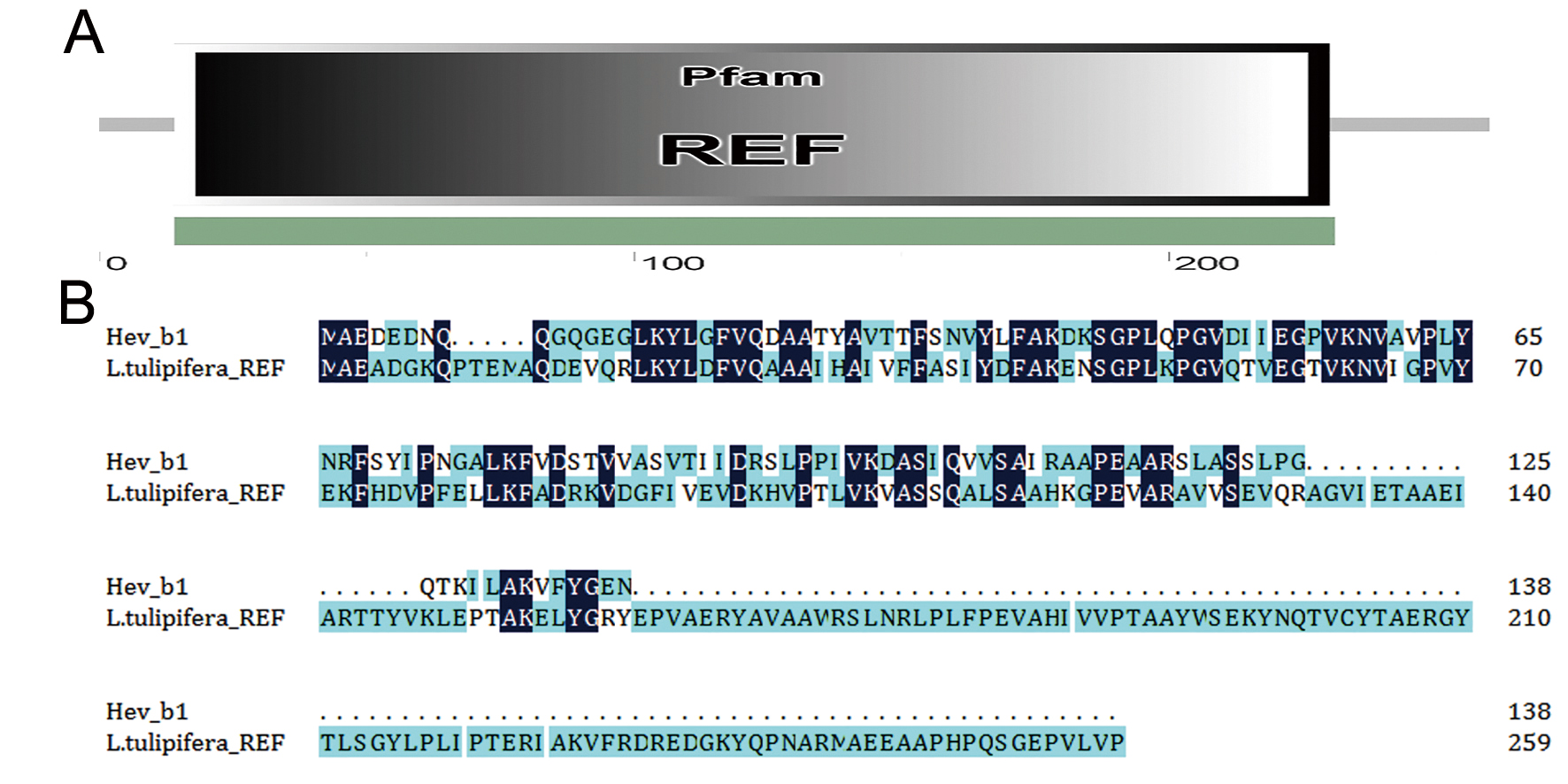

Supplement: Supplementary file 4 [file Image_1.JPEG]
